# Spatial distribution and the prevalence of speech disorders in the provinces of Iran 

**Published:** 2015

**Authors:** H Abbastabar, A Alizadeh, M Darparesh, S Mohseni, N Roozbeh

**Affiliations:** * Islamshahr Health and Treatment Network, Department of Health, Tehran University of Medical Sciences, Tehran, Iran; ** Department of Public Health, Faculty of Health, Hormozgan University of Medical Sciences, Bandar Abbas, Iran; *** Research Center for Social Determinants in Health Promotion, Department of Research and Technology, Hormozgan University of Medical Sciences, Bandar-e-Abbas, Iran; **** Department of Public Health, Faculty of Health, Hormozgan University of Medical Sciences, Bandar Abbas, Iran; ***** Reproductive Health, Shahid Beheshti University of Medical Science, Tehran, Iran

**Keywords:** developing countries, Iran, prevalence, spatial distribution, speech disorder

## Abstract

**Objective:** To identify the spatial distribution and prevalence of speech disorder in Iran.

**Materials and methods:** First, the prevalence of speech disorder in 2006 and 2011 was mapped via GIS for each province. Moreover, the prevalence of this disorder was calculated and classified according to age, sex, and residential area.

**Results:** The prevalence in the majority of provinces indicated an overall decrease. Furthermore, its prevalence among the whole population of Iran in 2006 and 2011 was 2.2 and 2 per 1000 people, respectively. The highest prevalence was observed among people aged 75 years and older. Results showed that speech disorders are more prevalent among men compared to women and also among rural residents compared to those in urban areas.

**Conclusion:** It is necessary to identify the high-risk areas in order to well organize the limited facilities to meet the actual needs of patients with speech disorder.

## Introduction

Speech disorders are communicative disabilities in which natural speaking faces difficulty. These disorders could be in the forms of stammering or lisping and so on. An individual afflicted with speech disorder is not able to produce perfect conversations. Due to speech problems, this person is considered to be dumb [**1**]. Classifying speech into “normal” and “disordered” is harder than it seems to be. According to precise classification attempts, only 5 to 10% of the world population are capable of speaking perfectly and produce proper speech sounds. The rest suffer from a sort of speech disorder [**1**,**2**].

Speech disorders are more common during childhood but they could possibly occur at any age. A recent research in Australia revealed that the occurrence of speech disorder among children at elementary school age and above could reach 13% [**3**]. In the majority of cases, the reason behind such disorders is unclear. However, among the known reasons are hearing loss, neural disorders, brain stroke, mental retardation, drug abuse, physical impairment such as cleft lip and palate, voice abuse and also child abuse [**4**].

In a systematic review study carried out in 2000 by Law et al., an average 5.95 % (range: 2.28-6.68) of children at the age of growth suffered from both speech disorders and language impairment. The estimated prevalence of language impairment was of only 2.02-19%. The prevalence range for speech disorder was merely 2.30-24.60 % [**4**]. McLeod % McKinnon reported that 13% of children at primary school and junior high school age were recognized by their teachers to suffer from speech disorders. The prevalence of communicative disorders was found to be higher than that of behavioral, mental, hearing and speaking disorders [**3**].

Harasty and Reed assessed the speaking ability of 437 nursery school children until the age of 6 in terms of speech and language impairments. 15.3% of the children were found to suffer from speech disorder while 20.6% of them had language impairment [**5**]. In Lawrence, the prevalence of delayed speech among children of 6 years of age was found to be 3.8%. The same prevalence, among boys, was observed to be 1.5 times as much as the girls (4.5% of the boys in contrast to 3.1% of the girls) [**6**]. The total number of the disabled in Iran in 1986 was of 453090 people. Those with speech disorder comprised 49032 of the population (27985 males and 21047 females). They were actually 10.8% of the whole population of the disabled. The most common reasons for the occurrence of speech disorder include hereditary factors, diseases, and accidents that comprised 61.8, 25.9, and 5.4% of all, respectively. In 2012, the number of the disabled due to speech disorders was 2.8 times as high, and reached 136829 people (81771 males and 55058 females). Considering the increasing number of the disabled up to 1271290, the ratio of people with speech disorder to the country’s total number of the disabled has remained rather unchanged and reached 10.76 percent [**7**].

Both genetic factors and environmental ones play a role in causing speech disorders. The effectiveness of these variables varies across different age, sex, and residential area groups [**7**]. On the other hand, finding out about the degree of occurrence of the disease along with its variation tendency plays a significant role in controlling the disease. According to the results of an investigation done by the authors of the present study, no proper research on speech disorders has been carried out in Iran so far. The range of information related to the occurrence and prevalence of this disease in the country is really incomplete.

However, the statistics and indices related to both occurrence and prevalence of such disorders are the key means of investigating them. The index of prevalence is essential for planning the healthcare service provided for these patients. The index of occurrence is vital for the etiologic research and the determination of the cause of the disease. The present research aimed to locate the spatial distribution of speech disorder in Iran during 2006 and 2011 in order to estimate their prevalence in different provinces of the country. It is actually a pioneering research in this realm, which helps a better healthcare provision for these patients.

## Methods

This descriptive research was done with the aim of determining the spatial distribution as well as the estimation of the prevalence of speech disorder in different provinces of the country. The data related to the whole population were obtained from the national center of statistics. The data related to speech disorder were obtained from the central welfare organization. The speech disorder data used in this research included all types of speech disorder. Briefly, disabled persons or their parents referred to the welfare organization and filled out an application form. Then they were investigated in a medical commission. If the commission verified their disabilities, then they would be divided into mild, moderate, severe, and greatly severe groups.

First, the degree of prevalence of speech disorders in 2006 and 2011 was mapped via GIS for each province. In order to estimate the prevalence of speech disorder in each province during these years, the frequency of speech disorder in each province was divided according to the total population of that province in that year. By means of estimating the degree of prevalence of this disorder for each province and via GIS, high-risk areas can be differentiated from low-risk areas of the country.

Moreover, the percentage of the speech disorder was estimated concerning age, sex, as well as the residential area. To do this, first, the participants were classified in the following age groups: 0-14, 15-29, 30-44, 45-59, 60-74 and above 75 years. In terms of the residential area, they were categorized as rural and urban. Concerning their gender, they were classified as male and female. Subsequently, the frequency of speech disorder in each category was divided by the sum of frequencies in each group. Furthermore, in order to estimate which percentage of the total speech disorder occurred in sex and residential area sub-categories, the total number of disabilities in these sub-groups (sum of columns), was divided by the total number of speech disorders. Finally, the prevalence of speech disorder was estimated according to age, sex, and residential area. In order to do this, the frequency of speech disorder in each sub-group was divided by the total number of population in that sub-group. Estimations were made via Microsoft Office Excel 2007. ArcMap 9.3 GIS software by ESRI was utilized to do the mapping.

**Ethics approval**

This study was approved by the ethical committee of welfare organization of Hormozgan province.

## Results

**Fig. 1:**
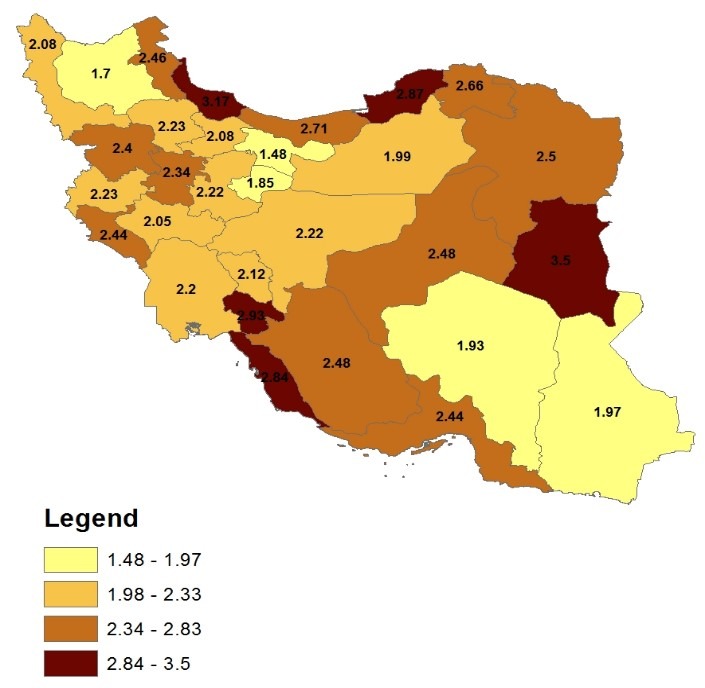
Prevalence of speech disorders during 2006 in the provinces of Iran

**Fig. 2:**
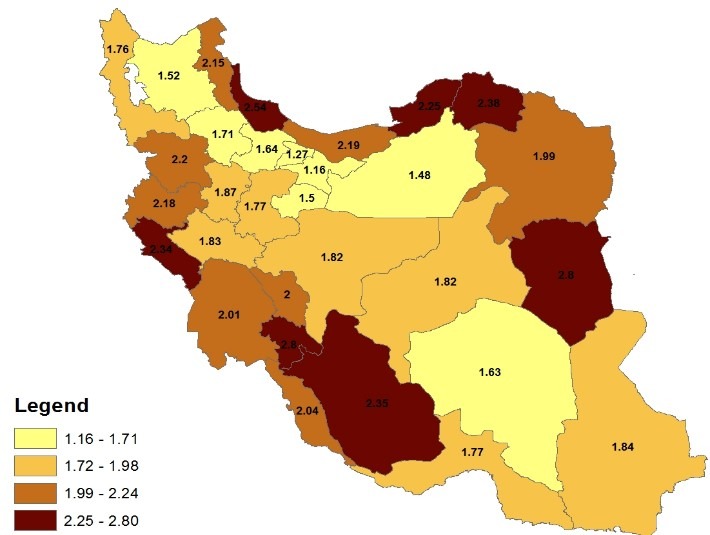
Prevalence of speech disorders during 2011 in the provinces of Iran

**Fig. 1** depicted the prevalence of speech disorder for all provinces of the country during 2006. Speech disorder was most prevalent in Bushehr, Kohkiloye va Boyer Ahmad, South Khorasan, Golestan and Gylan provinces. In East Azerbaijan, Tehran, Qom, Kerman and Sistan va Baluchestan it was the least prevalent. **Fig. 2** indicates that in 2011 this prevalence was the highest in Fars, Kohkiloye va Boyer Ahmad, Ilam, South and North Khorasan, Golestan and Gylan provinces, and the lowest in East Azerbaijan, Tehran, Qom, Karaj, Qazvin, Zanjan, Kerman and Semnan. Moreover, the estimates of the prevalence of speech disorder in the majority of Iran’s provinces showed the fact that the rate of this prevalence has been reduced during these years.

**Table 1** indicates the percentage of speech disorder in the subgroups of age, sex, and residential area in 2006 and 2011. The results in**Table 1A** reveal that in 2006, among urban and rural males, the highest percentages of disorder were 0.36 and 0.37 respectively, which occurred in the age range of 15-29. In the group of urban and rural females, the highest rates were 0.34 and 0.36, respectively, which occurred in the age range of 15-29. Moreover, among the total cases of speech disorder concerning sex and residential area, 0.36, 0.25, 0.23 and 0.16 of the cases were urban males, rural males, urban females and rural ones, respectively.

**Table 1B** is indicative of the results in percentage in 2011. In the group of urban and rural males, the highest rates were 0.32 and 0.35 respectively, which occurred in the age range of 15-29 years. Among urban and rural females, the highest rates were 0.31 and 0.34, which also occurred in the age range of 15-29. Moreover, among the total cases of speech disorder in the sex and residential area groups, 0.37, 0.23, 0.25 and 0.16 of the cases were urban males, rural males, urban females, and rural females, respectively.

**Table 1:** Proportion of speech disorders in the subgroups of age, sex and residence location during 2006 and 2011

**Table 1 A**. Proportion of speech disorders in the subgroups of age, sex and residence location during 2006										
Total		Female				Male				
		Rural		Urban		Rural		Urban		Age groups
Percent	Number	Percent	Number	Percent	Number	Percent	Number	Percent	Number	
0.26	39934	0.27	6400	0.25	8768	0.27	10133	0.26	14633	0 - 14
0.36	54424	0.36	8534	0.34	11886	0.37	14081	0.36	19923	15 - 29
0.18	27333	0.19	4532	0.18	6413	0.18	6891	0.17	9497	30 - 44
0.09	14483	0.09	2194	0.10	3511	0.09	3257	0.10	5521	45 - 59
0.07	9989	0.06	1400	0.07	2437	0.06	2310	0.07	3842	60 - 74
0.04	6445	0.04	922	0.05	1812	0.04	1441	0.04	2270	75 >
1	152608	0.16	23982	0.23	34827	0.25	38113	0.36	55686	Total
**Table 1 B **. Proportion of speech disorders in the subgroups of age, sex and residence location during 2011										
Total		Female				Male				
		Rural		Urban		Rural		Urban		Age groups
Percent	Number	Percent	Number	Percent	Number	Percent	Number	Percent	Number	
0.22	30590	0.22	4681	0.22	7464	0.22	7084	0.23	11361	0 - 14
0.33	44917	0.34	7248	0.31	10380	0.35	11170	0.32	16119	15 - 29
0.21	28615	0.23	4850	0.21	7097	0.21	6768	0.20	9900	30 - 44
0.12	17051	0.12	2613	0.13	4214	0.11	3544	0.13	6680	45 - 59
0.07	8990	0.06	1213	0.07	2443	0.05	1731	0.07	3603	60 - 74
0.05	6491	0.04	907	0.06	1885	0.04	1365	0.05	2334	75 >
1	136654	0.16	21512	0.25	33484	0.23	31662	0.37	49997	Total

**Table 2** is indicative of the prevalence of speech disorder in subgroups of age, sex, and residential area during 2006 and 2011 among 100 members of the population. The prevalence of the disorder in 2006 is presented in **Table 2A**. The highest prevalence rates were observed among urban and rural males: 0.0054 and 0.0050. It occurred in the age category above 75. In the subgroups of urban and rural females, these rates were 0.0044 and 0.0042, which also occurred in the age category above 75. In addition, in the last column of **Table 2A**, we can observe the prevalence of speech disorder in terms of age. The highest rate is 0.0048, which occurred in the age category above 75.The last row of Error! Reference source not found. showed the prevalence of speech disorder regarding sex and residential area. As it can be seen, the prevalence of disorder among urban males, rural males, urban females, and rural females is of 0.0023, 0.0034, 0.0015 and 0.0022, respectively. This prevalence was found to be 0.0022 in the whole population of Iran.

The prevalence of speech disorder in 2011 was indicated in **Table 2 B**. In the subgroups of urban and rural males, the highest rates were 0.0039 and 0.0041 respectively, which occurred in the age category above 75. In the subgroups of urban and rural females, the highest rates were 0.0030 and 0.0032 occurring in the age category above 75. As observed in the last column of **Table 2 B**, the prevalence of retardation was estimated with regard to age. The highest degree was found to be 0.0035, which occurred in the age category above 75. In addition, the last row of **Table 2 B** showed the prevalence of speech disorder with regard to sex and residential area. It was estimated to be 0.0021, 0.0035, 0.0013 and 0.0023 among urban males, rural males, urban females and rural females; respectively. The prevalence of speech disorder in the whole population of Iran was estimated to be 0.0020.

**Table 2:** Prevalence rate of speech disorders in the subgroups of age, sex and residence location during 2006 and 2011

**Table 2 A**. Prevalence of speech disorders in the subgroups of age, sex and residence location during 2006										
Total		Female				Male				
		Rural		Urban		Rural		Urban		Age groups
Percent	Number	Percent	Number	Percent	Number	Percent	Number	Percent	Number	
0.0007	39934	0.0021	6400	0.0016	8768	0.0032	10133	0.0026	14633	14 - 0
0.0022	54424	0.0022	8534	0.0014	11886	0.0034	14081	0.0023	19923	15 - 29
0.0019	27333	0.0023	4532	0.0012	6413	0.0034	6891	0.0018	9497	44 - 30
0.0027	14483	0.0018	2194	0.0012	3511	0.0030	3257	0.0018	5521	59 - 45
0.0026	9989	0.0022	1400	0.0021	2437	0.0034	2310	0.0030	3842	60 - 74
0.048	6445	0.0042	922	0.0044	1812	0.0050	1441	0.0054	2270	75 >
0.0022	152608	0.0022	23982	0.0015	34827	0.0034	38113	0.0023	55686	Total
**Table 2 B **. Prevalence of speech disorders in the subgroups of age, sex and residence location during 2011										
Total		Female				Male				
		Rural		Urban		Rural		Urban		Age groups
Percent	Number	Percent	Number	Percent	Number	Percent	Number	Percent	Number	
0.0023	30590	0.0023	4681	0.0015	7464	0.0035	7084	0.0024	11361	0 - 14
0.0023	44917	0.0026	7248	0.0014	10380	0.004	11170	0.0024	16119	15 - 29
0.0016	28615	0.002	4850	0.001	7097	0.003	6768	0.0014	9900	30 - 44
0.0014	17051	0.0017	2613	0.0009	4214	0.0027	3544	0.0014	6680	45 - 59
0.0023	8990	0.002	1213	0.0016	2443	0.0039	1731	0.0026	3603	60 - 74
0.0035	6491	0.0032	907	0.003	1885	0.0041	1365	0.0039	2334	75 >
0.002	136654	0.0023	21512	0.0013	33484	0.0035	31662	0.0021	49997	Total

## Discussion

Findings of the present research are indicative of a decreasing trend in the prevalence of speech disorders during the recent years in Iran. Speech disorders were found to be the most prevalent in rural residential areas among males above 75 years of age.

The comparison of the prevalence of speech disorders in 2006 and 2011 revealed that their degree of prevalence has decreased during the recent years. Findings of a longitudinal study by ASHA indicated that the total number of speech and language pathologists of the U.S.A. has consistently increased during the past 10 years. In 1989, they were 57167 in number. Between 1989 and 1999, 41000 new experts were added. In 1999, this number reached 98522 [**8**]. The decrease in the prevalence of speech disorders in Iran could also be due to its recent medical advancements. Due to such an improvement, some types of speech disorders, which were previously incurable and patients had to live with them until the end of their lives, can now be treated. On the other hand, the decrease in the prevalence could also be due to the diagnosis of minor speech disorders which were not diagnosable in the past, but can now be diagnosed through highly advanced medical techniques

The prevalence of all types of speech disorders in Iran has been reported in the present research to be 2.2 per a thousand people in 2006, and 2 per a thousand people in 2011. In a study carried out by Tomblin et al. on 6-year-old children, the prevalence of language impairment and speech sound disorder was found to be 7.4% and 8.2% respectively [**9**]. In another study by Sheriberg, the prevalence of delayed speech was observed to be 3.8%. Moreover, in this study, such prevalence was found to be 4.5% among boys and 3.1% among girls [**6**]. In a research conducted by Bietchman et al. on 5-year-old children, 1655 children were participating. 180 of them were diagnosed to have language impairment or speech sound disorder. In this study, the prevalence of speech disorder was estimated to be 16.2 to 21.8% [**10**]. The reason why there seems to be a divergence between the prevalence found in our study with those of the previously mentioned research could be among the following. Firstly, our study was conducted on all types of speech disorders while each of the previous research was focused on a particular type. Secondly, in our study, the prevalence was found to be, for the whole population of all age groups, of approximately 2 per a thousand people. However, in the other body of research, the prevalence was estimated only for 6-year-old children.

Findings, both as proportions and as prevalence, indicated that the percentage of speech disorders was highest among rural people, and also among the male rather than the female. According to the study conducted by Sheriberg, the prevalence of delayed speech among children was found to be 1.5 times as much as the boys [**6**].In another study carried out by Bietchman et al., the prevalence of speech disorders was estimated to be 15.5% to 20.7% among boys and 19.1% to 25.1% among girls [**10**]. Concerning the contradiction observed between the degrees of prevalence in terms of sex, we could say that firstly, these divergences could be actual. Sex-related distribution of etiologic factors of speech disorder differs from one population to another. Secondly, this divergence could be due to sampling bias, that is, the sample selected for the study is not representative for the real population of adults or children. No research was done to take into account the residential area (rural vs. urban) as related to the prevalence of speech disorders. Concerning the higher prevalence of speech disorder in rural areas it can be said that first of all, the prevalence of common causes of speech disorders such as brain stroke [**5**,**6**] due to careers including agriculture and animal raising in these areas is higher in rural areas than in urban ones. Secondly, people living in rural areas have less access to medical healthcare facilities. Some of their minor speech disorders are curable. However, they are left untreated for the reason just discussed and they remain with patients all throughout their lives.

Findings in proportions revealed that the highest percentage of speech disorders occurred at the age range of 15-29. Findings in prevalence, however, revealed that both among males and females, such disorders were more prevalent at the age above 75. The majority of studies on speech disorders were conducted on children [**6**,**8**,**9**]. No study investigated all the age groups. Concerning the divergences observed between findings in proportion with those in prevalence, it could be said that the numerators of the fractions (in proportions) are similar to the prevalence values and they target all the people with speech disorders in that age group. The denominators of the fractions, however, consist of all the people suffering from speech disorders in all age groups. The denominator of the prevalence fraction, on the other hand, includes all the population at that age. Since the denominator is constant in all age groups, and usually in all age groups the highest percentage of population live between 15 to 29 years old, the numerator of the proportion fraction being increased in this age group compared to that of the others. 

## Conclusion

Since speech disorders include a wide range of disorders and in the majority of cases engage people from early school age, the determination of their geographical distribution is essential. Among the possible ways of reducing the occurrence, prevalence and rehabilitating the people afflicted with a kind of speech disorder are identifying high-risk regions, identifying social variables affecting speech disorders and organizing the use of the limited sources and facilities in order to meet the actual needs of patients with speech disabilities through focusing on three levels of prevention.

**Acknowledgements**

This study was supported by the state welfare organization of Hormozgan province.
